# Diabetes Mellitus Is a Strong Independent Negative Prognostic Factor in Patients with Brain Metastases Treated with Radiotherapy

**DOI:** 10.3390/cancers15194845

**Published:** 2023-10-04

**Authors:** Seong Jeong, Soniya Poudyal, Sabine Klagges, Thomas Kuhnt, Kirsten Papsdorf, Peter Hambsch, Johannes Wach, Erdem Güresir, Franziska Nägler, Alexander Rühle, Nils H. Nicolay, Clemens Seidel

**Affiliations:** 1 Department of Radiation Oncology, University of Leipzig Medical Center, 04103 Leipzig, Germanysonya95ger@gmail.com (S.P.); thomas.kuhnt@medizin.uni-leipzig.de (T.K.);; 2 Comprehensive Cancer Center Central Germany, Partner Site Leipzig, 04103 Leipzig, Germany; johannes.wach@medizin.uni-leipzig.de (J.W.);; 3 Clinical Cancer Registry, 04103 Leipzig, Germany; sabine.klagges@medizin.uni-leipzig.de; 4 Department of Neurosurgery, University of Leipzig Medical Center, 04103 Leipzig, Germany

**Keywords:** brain metastasis, radiotherapy, systemic therapy, diabetes mellitus, vascular risk factors, vascular comorbidities, frailty, arterial hypertension, smoking, hypercholesterolemia

## Abstract

**Simple Summary:**

Cerebrovascular disorders are common among cancer patients. They might influence tumor growth, treatment sensitivity and, ultimately, the prognoses of patients with brain metastases (BM). In a retrospective exploratory study, we examined if the presence of arterial hypertension, smoking, diabetes mellitus (DM), hypercholesterolemia or peripheral arterial occlusive disease has a prognostic impact in patients with BM. In uni- and multivariate analysis, the presence of DM was associated with a worse prognosis across several tumor types, while for the other cerebrovascular risk factors, significant differences in survival were not found. From molecular data, it can be hypothesized that RAGE activation plays an important role in the interaction between DM and BM. In future studies, it remains to be determined to what extent serum glucose levels and antidiabetic treatments may influence survival and if optimized antidiabetic treatment or RAGE targeted treatments are able to improve prognoses of patients with BM.

**Abstract:**

Background: Brain metastases (BM) cause relevant morbidity and mortality in cancer patients. The presence of cerebrovascular diseases can alter the tumor microenvironment, cellular proliferation and treatment resistance. However, it is largely unknown if the presence of distinct cerebrovascular risk factors may alter the prognosis of patients with BM. Methods: Patients admitted for the radiotherapy of BM at a large tertiary cancer center were included. Patient and survival data, including cerebrovascular risk factors (diabetes mellitus (DM), smoking, arterial hypertension, peripheral arterial occlusive disease, hypercholesterolemia and smoking) were recorded. Results: 203 patients were included. Patients with DM (*n* = 39) had significantly shorter overall survival (OS) (HR 1.75 (1.20–2.56), *p* = 0.003, log-rank). Other vascular comorbidities were not associated with differences in OS. DM remained prognostically significant in the multivariate Cox regression including established prognostic factors (HR 1.92 (1.20–3.06), *p* = 0.006). Furthermore, subgroup analyses revealed a prognostic role of DM in patients with non-small cell lung cancer, both in univariate (HR 1.68 (0.97–2.93), *p* = 0.066) and multivariate analysis (HR 2.73 (1.33–5.63), *p* = 0.006), and a trend in melanoma patients. Conclusion: DM is associated with reduced survival in patients with BM. Further research is necessary to better understand the molecular mechanisms and therapeutic implications of this important interaction.

## 1. Introduction

Brain metastases (BM) are frequent complications in patients with cancer. According to a SEER database analysis among patients with metastatic disease, patients with melanoma (28.2%), lung adenocarcinoma (26.8%), small cell lung cancer (23.5%), squamous cell carcinoma of the lung (15.9%), renal cancer (10.8%) and breast cancer (7.6%) had brain metastases [1]. Importantly, the incidence of brain metastases (BM) is increasing, partially due to longer life expectancy and better means of detection [2,3].

Despite more available treatments for BM, including surgery, stereotactic radiotherapy (SRT), whole-brain radiation therapy (WBRT), molecularly targeted therapeutics and immunotherapies, patients with BM mostly still have a poor prognosis [1,3,4,5,6].

However, in recent years, several radiotherapeutic developments have improved the treatment of brain metastases. One main focus of research was the prevention of cognitive decline that is frequent after conventional whole-brain radiotherapy (WBRT) [7,8]. Firstly, it has been shown in a multicentric observational trial that stereotactic radiotherapy can be applied to patients with 5–10 brain metastases without worse overall survival compared to patients with 2–4 BM [9,10]. In another approach to maintaining cognitive functioning, hippocampal sparing WBRT has shown significant benefits compared to conventional WBRT in a phase 2 and comparative phase 3 trial [11,12]. Within these trials, in combination with WBRT, the partial N-Methyl-D-Aspartate (NMDA)-receptor antagonist memantine was applied in order to further preserve cognitive function after WBRT [12,13].

Prognostic scores for patients with BM developed from disease-unspecific scores, like recursive partitioning analysis (RPA), to more disease specific approaches, like the disease-specific graded prognostic assessment (ds-GPA) score [14,15]. The ds-GPA encompasses tumor specific variables, like mutation status, extracranial tumor control and number of brain metastases, as well as some patient variables like patient age and Karnofsky performance score (KPS) [16]. Except for KPS, patient frailty and comorbidities are not reflected in the ds-GPA.

The concept of patient frailty comprises different parameters that better describe patient’s well-being, physical independency and comorbidities. Measures of frailty include G8 and Hurria Score [17,18]. Within general oncology, considerable knowledge has been gained regarding the relevance of comorbidities in prognoses and treatment of colorectal cancer, breast cancer and lung cancer [19,20,21]. However, comorbidities, as a part of the frailty concept, have not been a scientific focus in patients with BM so far.

Among co-morbidities, vascular conditions or risk factors might be particularly relevant in a specific and non-specific fashion. Firstly, and specifically, BM heavily rely on and interact with cerebral blood vessels as part of their microenvironment. Alterations in cerebral vasculature might modulate the number and growth of BM, as well as the sensitivity to therapeutic interventions like radiotherapy, which relies on appropriate perfusion and oxygenation [22,23,24].

Non-specifically, cerebrovascular comorbidities (diabetes mellitus (DM), smoking, arterial hypertension, peripheral arterial occlusive disease (PAOD), hypercholesterolemia) are frequent in cancer patients and there are ample indications that they may negatively affect the course of malignant disease.

The prevalence of hypertension is greater in cancer patients and survivors compared with the general population, and arterial hypertension carries a risk of multiple cardiovascular complications during cancer treatment and potentially increased mortality [25,26].

Smoking is associated with a poorer prognosis in patients with small-/non-small cell lung cancer (SCLC/NSCLC) and breast cancer [27,28,29]. Regarding hypercholesterolemia, cholesterol-lowering medication was associated with a decrease in cancer mortality in in a large meta-analysis of breast cancer patients [30]. Finally, diabetes mellitus (DM) appears to be associated with increased cancer mortality across several primary tumors [31,32].

The aim of this exploratory retrospective study was to examine the prognostic role of frequent cerebrovascular comorbidities, specifically in patients with BM undergoing radiotherapy.

## 2. Materials and Methods

This study was performed according to the Declaration of Helsinki in its current form. Approval was granted by the Ethics Committee of University of Leipzig (Date 3rd of August.2021/No332/21-ek). All patients consented to the anonymized scientific use of their clinical data. Patients treated with radiotherapy at the Department of Radiation Oncology, University of Leipzig Medical Center, Leipzig, Germany, between 2004 and 2016 were identified from clinical records. All patients (age ≥ 18 years) diagnosed with BM from a solid primary tumor were principally eligible for the study; consecutive patients were chosen with available pre-therapeutic cerebral MRI and detailed clinical patient charts. Treatment was either performed with SRT or WBRT, depending on number of metastases and patient conditions. Patient data were analyzed from existing patient charts. Various characteristics including age (at primary BM diagnosis), primary tumor type, KPS, number of metastases, systemic tumor control and the presence or absence of DM, arterial hypertension, smoking status, hypercholesterolemia, and PAOD were recorded prior to treatment of BM in a standardized fashion. Survival data were obtained from the local cancer registry. Data analysis was performed using Microsoft Excel 2016 (Microsoft Corporation, Albuquerque, NM, USA) and SPSS Version 28.0.1.1 (IBM Software Inc., Armonk, New York, USA). Overall survival (OS) was calculated from the date of diagnosis of brain metastasis until death; patients being lost to follow-up were censored at the last date known to be alive. OS was examined using Kaplan–Meier analyses with log-rank tests. Univariate and multivariate Cox proportional hazard regression analyses were conducted to reveal prognostic parameters associated with OS. For Cox proportional hazard regression analyses of established prognostic factors age was stratified to ≥70 years vs. <70 years, KPS <70 vs. ≥ 70 and number of BM >3 vs. ≤3 BM.

## 3. Results

### 3.1. Patient Characteristics

Data from 203 patients were included in the analysis. Patient age at diagnosis of BM ranged from 30 to 83 years with a median/mean age of 62.6/61.3 years. At diagnosis of BM, patients presented with a median Karnofsky performance status (KPS) of 70 (range: 20–100). The most common primary tumor was non-small cell lung cancer (NSCLC) in 84/203 patients (41.4%), followed by melanoma (28/203, 13.8%), breast cancer (21/203, 10.3%), small cell lung cancer (SCLC) and renal cell carcinoma (RCC) (each 20/203, 9.9%), colon cancer (7/203, 3.4%), and other cancers (23/203, 11.3%). Concomitant vascular co-morbidities were arterial hypertension (100/203 patients, 49.3%), DM (39/203, 19.2%), peripheral arterial occlusive disease (PAOD) (23/203, 11.3%), hypercholesterolemia (7/203, 3.5%). In total, 33.5% of patients were smokers (68/203 patients, Table 1), 39.4% (80/203) of patients were diagnosed with 1–3 BM, and 90 patients (44.3%) had >3 BM. The majority of patients had unstable systemic disease or synchronous brain metastases (137/203, 67.4%). Treatment of patients BM comprised stereotactic radiotherapy (SRT) (40/192, 19.7%) and whole-brain radiotherapy (WBRT) ± SRS (152/191, 74.8%).

### 3.2. Age, KPS and Tumor Histology Influenced Survival Outcomes

The median survival time of the entire cohort was 6 months (95% CI: 4.49–7.40), and the median follow up was 6.01 months (95% CI: 0.93–42.54). Median survival times were different between patients with age ≥70 years/<70 years (4.14/7.29 months, *p* = 0.001) and with KPS <70, ≥70 (3.06/6.70 months, *p* = 0.03) but not between patients with >3, ≤3 BM: 5.55/6.74 months (*p* = 0.443) respectively. Concerning tumor histology, survival times for patients were as follows: NSCLC, 6.01 months; SCLC, 5.52 months; melanoma, 7.46 months; breast cancer, 9.53 months; RCC, 2.96 months; and other cancers, 4.17 months.

In the univariate Cox regression analysis, patients age ≥70 years showed significant detrimental effects regarding survival (HR 1.75 [1.24–2.48], *p* = 0.002). Furthermore, KPS <70 and tumor histology (RCC) were associated with reduced survival (KPS <70: HR 1.47 [1.03–2.1], *p* = 0.033, RCC: HR 2.035 [1.26–3.28], *p* = 0.015). The number of BM was not associated with differences in survival time (>3 BM: HR 1.13 [0.83–1.54], *p* = 0.444; Table 2).

### 3.3. Diabetes Mellitus Was Associated with the Reduced Survival of Patients with Brain Metastases Undergoing Radiotherapy

Median survival times for patients with/without DM, with/without arterial hypertension, with/without smoking, with/without PAOD, and with/without hypercholesterolemia were 4.73/6.7 months (*p* = 0.003), 5.95/6.05 months (*p* = 0.443), 6.08/5.72 months (*p* = 0.307), 4.17/5.95 months (*p* = 0.266), and 4.11/5.71 months (*p* = 0.157), respectively.

**Figure 1:**
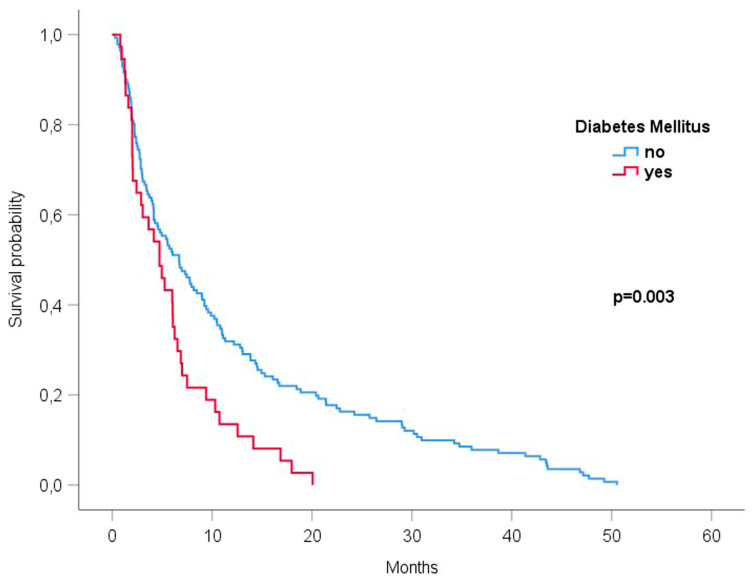
Survival after diagnosis of BM (Kaplan–Meier analysis and log-rank test).

In the Kaplan–Meier analysis, patients with DM had significantly shorter survival compared to patients without DM (HR 1.75 [1.20–2.56], *p* = 0.003, Figure 1).

**Figure 2:**
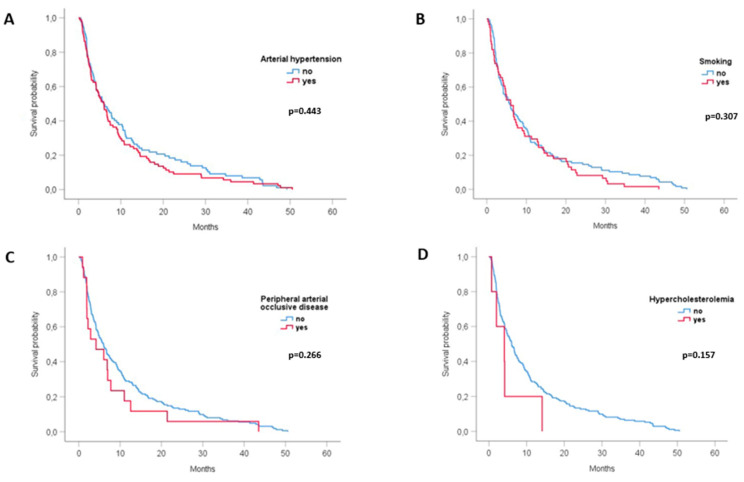
Survival according to vascular comorbidity (Kaplan–Meier analysis and log-rank test). (**A**). Survival propability of patients with (red)/without (blue) arterial hypertension, (**B**). Survival propability of smokers (red)/non-smokers (blue), (**C**). Survival propability of patients with (red)/without (blue) peripheral arterial occlusive disease, (**D**). Survival propability of patients with (red) /without (blue) hypercholesterolemia.

The presence or absence of other vascular comorbidities was not associated with differences in survival (Table 2, Figure 2A–D).

Patients with and without DM were compared to investigate potential confounders. Patients without DM were younger (*p* = 0.027), systemic tumor progression at time of diagnosis of BM appeared somewhat but was insignificantly more frequent in patients with DM (19.4% vs. 5.7%, *p* = 0.154). Otherwise, the distribution of KPS (*p* = 0.841), histology (*p* = 0.606), number of BM (*p* = 0.792) and radiotherapy concept (*p* = 0.441) were not different, a shown in Table 3.

### 3.4. Diabetes and Patient Age Are Independent Negative Prognostic Factors in Patients with Brain Metastases Undergoing Radiotherapy

Multivariate Cox regression analysis was performed, including the following factors: DM, age (<70, ≥70), KPS score (<70, ≥70), and number of BM (≤3; >3).

**Figure 3:**
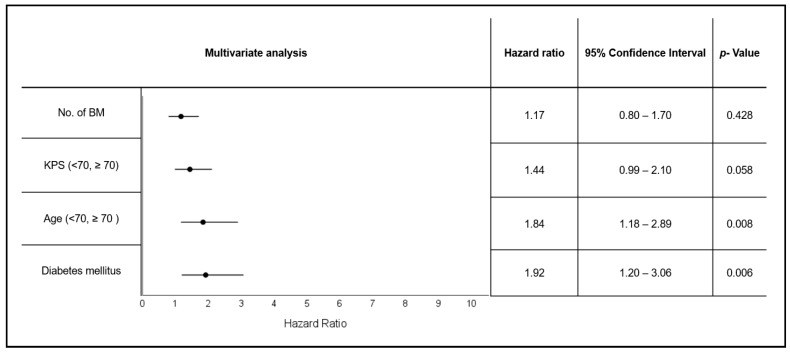
Multivariate analysis of survival, including relevant prognostic factors.

DM (HR 1.92 [1.20–3.06], *p* = 0.006, Figure 3) and age ≥70 (HR 1.84 [1.18–2 89], *p* = 0.008) remained independently associated with worse OS. KPS <70 showed a trend (HR 1.44 [0.99–2.1], *p* = 0.058) towards deteriorated OS. No effect was seen for the number of BM (HR 1.17 [0.8–1.70], *p* = 0.428), Figure 3.

### 3.5. Diabetes Mellitus Is a Negative Prognostic Factor for Brain Metastases of Distinct Histologies

In order to further validate the negative prognostic impact of DM, univariate and multivariate analyses were performed separately for the two most common primary cancer types in our cohort, i.e., NSCLC (84 patients) and melanoma (28 patients). In patients with NSCLC, the presence of DM was associated with poorer survival in the univariate (HR 1.68 [0.97–2.93], *p* = 0.066) and multivariate analysis (HR 2.73 [1.33–5.63], *p* = 0.006, Supplementary Figure S1). Age as an established important factor lost significance in the analysis restricted to NSCLC (univariate: HR 1.54 [0.85–2.72], *p* = 0.151; multivariate: HR 1.31 [0.63–2.73], *p* = 0.475). The negative impact of lower KPS persisted (univariate: HR 2.47 [1.40–4.38], *p* = 0.002; multivariate: HR 2.85 [1.52–5.33], *p* = 0 001, Supplementary Table S1).

In the much smaller cohort of melanoma patients, a worse survival with DM appeared from survival curves; however, in uni- and multivariate Cox regression, this was not significant (multivariate: HR 4.62 [0.49–44.04], *p* = 0.183), and other factors were not associated with differences in survival (Supplementary Figure S2 and Supplementary Table S2).

## 4. Discussion

BM are a “special” complication of cancer in a special organ, often requiring specific treatments. Accordingly, aspects like metastasis formation, metastasis growth, treatment, resistance to treatment and prognosis need to be specifically addressed.

The formation of BM depends on the extravasation of tumor cells, perivascular tumor cell growth, and the co-option of pre-existing vessels in a complex multistep process [33,34,35]. In addition, cerebral perfusion and oxygenation are crucial for the effect of local radiotherapy on BM [36,37], and alterations in cerebral perfusion could also modulate the efficacy of systemic treatment [38,39]. Taking into account this relevant interplay of BM formation and treatment with vascular architecture, there is a strong need to more precisely understand the effects of vascular risk factors that could influence development and prognosis of BM. Cerebrovascular risk factors are frequent and often lead to chronic cerebrovascular diseases [40,41]. Macro- and microvascular changes predispose to multiple complications, including vascular stenosis, ischemic strokes, and small vessel disease of the brain [42,43].

Within this exploratory study, we examined if several frequent cerebrovascular risk factors may have a prognostic effect in patients with BM treated with radiotherapy. Arterial hypertension, smoking, PAOD, and hypercholesterolemia had no prognostic value both in monovariate and multivariate analysis. Based on chart-based diagnoses in this moderately sized cohort, this does not exclude minor individual effects, but a larger effect of these frequent cerebrovascular risk factors in patients with BM seems unlikely. Coherent with our results, a large retrospective study in 390 patients with lung cancer and BM, concerning the effect of smoking, found that smoking status and pack-year history of smoking had no effect on overall survival; a trend for an increased risk of neurologic death in non-adenocarcinoma patients who continued to smoke was postulated [44].

Beyond the functional effects of risk factors, existing structural vascular alterations that have not been measured in this patient cohort might be more important.

It is known that small vessel disease of the brain can influence the number of BM, potentially by reducing the accessibility of the less perfused brain areas [45,46,47].

The relevance of small vessel disease of the brain in patient prognosis should be examined in future studies. The same is true for the presence of angiopathic alterations in large cerebral vessels and history of ischemic vascular events/strokes.

In contrast to the other mentioned vascular risk factors, DM showed a strong independent negative prognostic effect.

From clinical evidence, little is known about the prognostic effects of DM specifically in patients with BM treated with radiotherapy thus far. In a smaller single-center retrospective study, 81 patients with BM from breast cancer who were treated with stereotactic radiation therapy were retrospectively analyzed regarding a prognostic effect of obesity and DM. Patients with DM (*n* = 17) had decreased median OS (11.8 vs. 26.2 months; *p* < 0.001) and median intracranial PFS (4.5 vs. 10.3 months; *p* = 0.001) compared to non-diabetic patients (*n* = 67). On multivariate analysis, both BMI ≥ 25 kg/m^2^ [HR 2.35 (1.39–3.98); *p* = 0.002] and diabetes (HR 2.77 [1.454–5.274]; *p* = 0.002) were associated with increased mortality [48].

A second retrospective single-center analysis of a larger cohort of patients with various primary tumors (498 patients/48 patients with DM) reported results for stereotactic radiotherapy [49]. DM was found to be a significant negative predictor of OS on multivariate analysis (HR: 1.41, CI: 1.02–1.95, *p* = 0.04).

Concurring with these two studies, our data support DM as an independent factor associated with mortality in patients with BM. In the series by McCall and colleagues, only patients with breast cancer were included, and in the work of LeCompte, several tumor histologies with a focus on NSCLC were included [48,49]. Together with our results, a tumor-independent effect concerning patients with disseminated malignancies is most likely. With a HR of 1.92, our results appear to be within the range of those from the cited studies: 1.4–2.77 [48,49]. The negative effect appears to persist in different age groups and treatment scenarios (McCall: younger patients with 80% WBRT [48], LeCompte: SRS only [49]).

Concerning the reasons for a negative prognostic impact, both series contain valuable information. While in the study by LeCompte, the major detriment appeared to arise from systemic effects (death prior to distant brain failure earlier in diabetics vs. non-diabetics (*p* = 0.04), in the series by McCall, median intracranial PFS was significantly reduced in patients with DM [48,49]. Our study did not plan to analyze PFS but was restricted to OS without providing reasons for death. Notably, we aimed to examine a patient cohort treated with radiotherapy to BM prior to the widespread use of systemic treatments effective in BM. With the more frequent use of immune checkpoint inhibitors or targeted treatments, the prognostic relevance of DM in patients with BM might be less pronounced and should also be examined in these patient cohorts.

We did not examine potential associations between DM and treatment-related toxicities. However, the study of LeCompte et al. did not observe significant differences in the incidence of radiation necrosis, radiation-induced edema, cerebrospinal fluid leak or postoperative infection in patients with DM [49].

While clinical evidence of the negative effects of DM in BM is limited, it is better known for its negative effects on cancer patients with regard to different primary tumors. DM contributes to increased mortality from colorectal cancer, liver cancer, pancreatic cancer, breast cancer, or lung cancer [22,50,51].

Several pathophysiologic aspects of diabetes mellitus type 2 interact with tumor metabolism. According to clinical and preclinical evidence, hyperinsulinemia, hyperglycemia and a diabetes-associated chronic inflammatory state appear to be associated with elevated cancer risk and mortality [50,52]. In a large prospective cohort study with around 10,000 participants, hyperinsulinemia, even without manifest diabetes mellitus, was associated with increased cancer mortality [53]. In a preclinical model, hyperinsulinemia promoted metastasis in the lungs in a mouse model of Her2-mediated breast cancer [54]. Hyperglycemia itself leads to growth promotion and increased proliferation as tumors mostly rely on anerobic glycolysis [55]. Glycolysis is facilitated by upregulated glucose transporters (GLUTs) in tumor cells, enabling the facilitative entry of glucose into a cell. A high rate of glucose can be consumed by malignant cells beyond that necessary for ATP synthesis [56,57].

Hyperglycemia is thought to play a key role in tumor progression by reprogramming glucose metabolism, stimulating cancer-associated inflammation, molecular alterations and hypoxia [58,59,60,61]. It can also lead to therapeutic resistance through immunosuppression [62,63], which contributes to poor outcomes in tumor patients [64].

**Figure 4:**
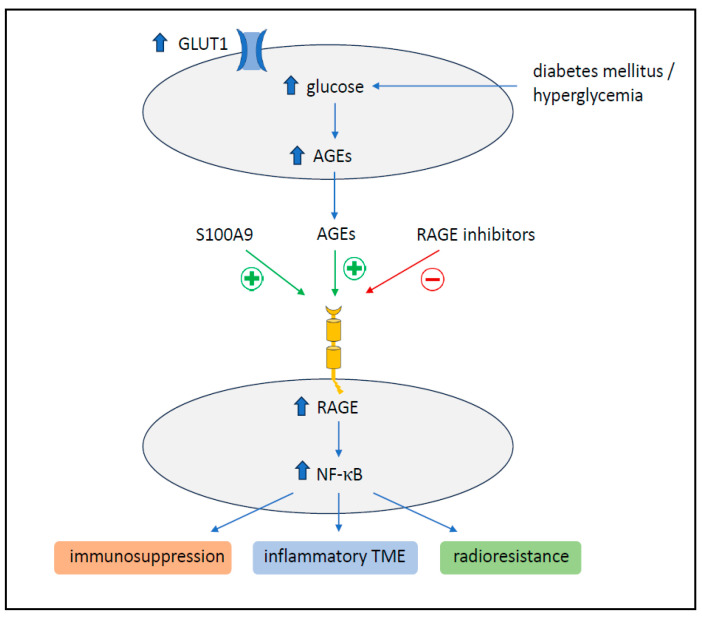
Role of RAGE activation in BM and DM; RAGE can be activated by several mechanisms including hyperglycemia and, via NF-ҝB, contributes to immunosuppression as well as local tumor microenvironment and, eventually, the radioresistance of BM. RAGE activation can be reversed by specific receptor inhibitors.

An important mechanism in the interplay of diabetes mellitus and malignant disease is the overactivation of the receptor for advanced glycation end-products (RAGE). RAGE is activated as a consequence of the increase in glycolysis, which enhances the non-enzymatic glycation of proteins, leading to the formation of advanced glycation end-products (AGEs) [55]. AGEs, particularly N-carboxymethyllysine [CML]-modified proteins, were the first RAGE ligands to be identified [65]. The overexpression and activation of RAGE is able to continuously fuel an inflammatory milieu in the tumor microenvironment [66,67]. Interestingly, the overactivation of RAGE as part of the S100A9-RAGE-NF-κB-JunB pathway was discovered as a mechanism for the radioresistance of brain metastasis [68]. Brain metastatic cancer cells from different primary tumors were found to highly express S100A9 in the brain microenvironment, mediating resistance to radiotherapy via the downstream activation of RAGE and NF-κB. S100A9 expression in human brain metastasis from patients with lung cancer, breast cancer or melanoma negatively correlated with the benefits of radiotherapy. The genetic or pharmacological targeting of S100A9 via a blood–brain-barrier-permeable inhibitor of its receptor (RAGE) sensitized brain metastasis to irradiation in experimental models of brain metastasis as well as in patient-derived organotypic cultures [68]. Together with other data concerning DM, it can be suggested that DM/hyperglycemia might confer radioresistance via RAGE activation—potentially independently from S100A9—but might be reversed by RAGE inhibitors too (Figure 4). A clinical trial exploiting the mechanism of RAGE inhibition as a means of radiosensitisation is currently planned [69].

As a further molecular crosslink of altered glucose metabolism and treatment of BM, a recent multiplex immunofluorescence study of resected BM samples from 33 patients treated with radiotherapy and ipilimumab for BM of melanoma found a strong upregulation of the glucose transporter GLUT 1 in BM associated with a prognostic detriment in these patients [70].

It can further be speculated that DM or hyperglycemia is particularly relevant in BM due to the constantly high perfusion of the brain and frequent use of corticosteroids for the alleviation of neurologic symptoms. In other brain tumors like malignant glioma, a significant prognostic deterioration through the use of dexamethasone was shown in large pooled analysis [71].

From the existing data, both a systemic and local tumor-growth-promoting effect appear likely, while alterations of cerebral blood vessels seem less plausible as a cause of shortened survival.

Our analysis carries the limitations of a monocentric retrospective series with potential confounders. We tried to control many factors using a multivariate analysis, and the frequency of several other factors like treatment type and the state of systemic tumor control was not significantly different between patients with and without DM. However due to the limited sample size, not all effects may have been detected.

Several important questions remain from ours and other studies. Firstly, a potential direct negative correlation between survival time and serum glucose levels needs to be analyzed further, as well as the potential effect of antidiabetic treatments, most preferably in a prospective multicentric fashion. In our cohort, repeated serum glucose levels were not consistently available. Secondly, dexamethasone use in patients with BM should be monitored and correlated to blood glucose levels and outcomes. Furthermore, much more specific research is needed to characterize diabetes-specific changes in brain metastasis that might be prognostically and therapeutically relevant. In particular, a large-scale comparative analysis between tumors/brain metastasis in patients with and without DM should be performed to better understand biologic and prognostic differences. In the future, prognostic scores for BM should most likely be expanded to include DM and potential other factors.

## 5. Conclusions

DM seems to negatively affect survival time, but we did not find any general effect of smoking, arterial hypertension, PAOD, and hypercholesterolemia on overall survival in BM (multi-center validation pending). DM appears as a strong independent risk factor for growth, microenvironment, and therapy resistance, probably not related to vascular effects but more likely to pleiotropic effects. The correlation of blood serum levels to outcomes and potential therapeutic implications (e.g., of RAGE inhibition) need to be evaluated in patients with BM in future.

For now, the presence of DM appears potentially more relevant for patients’ survival times than the choice of radiotherapy concept [72,73]. The focus of treating radiation oncologists needs to shift more closely to this potentially influenceable patient condition.

## Figures and Tables

**Table 1:** Patients’ characteristics.

Characteristic	Group	Patients *n* = 203 (%)
Age	<70 years	154 (76)
	≥70 years	49 (24)
KPS	≥70	104 (51.2)
	<70	52 (25.6)
	Missing data	47 (23.2)
No. of brain metastases	>3	90 (44.3)
	≤3	80 (39.4)
	Missing data	33 (16.3)
Tumor type	NSCLC	84 (41.4)
	SCLC	20 (9.9)
	Breast cancer	21 (10.3)
	Melanoma	28 (13.8)
	RCC	20 (9.9)
	Other	30 (14.7)
Stable systemic disease	Yes	27 (13.3)
	No	71 (34.9)
	Synchronous BM	66 (32.5)
	Missing data	39 (19.2)
Radiotherapy technique	SRT	40 (19.7)
	WBRT ± SRT	152 (74.8)
	Missing data	11 (5.5)
Diabetes mellitus	Yes	39 (19.2)
	No	164 (80.3)
	Missing data	0 (0)
Arterial hypertension	Yes	100 (49.3)
	No	94 (46.3)
	Missing data	9 (4.4)
Smoking	Yes	68 (33.5)
	No	128 (63.1)
	Missing data	7 (3.4)
Peripheral arterial occlusive disease	Yes	23 (11.3)
	No	176 (86.7)
	Missing data	4 (2)
Hypercholesterolemia	Yes	7 (3.5)
	No	189 (93)
	Missing data	7 (3.5)

**Table 2:** Univariate analysis of survival in all patients.

Characteristic	Median OS	*p*-Value (Log Rank)
Age		0.001
<70 years	7.29 (5.34–9.24)	
≥70 years	4.14 (2.43–5.85)	
KPS		0.03
≥70	6.70 (2.99–10.42)	
<70	3.06 (1.16–4.95)	
No. of brain metastases		0.443
>3	5.55 (4.03–7.07)	
≤3	6.74 (4.63–8.84)	
Tumor type		0.02
NSCLC	6.01 (3.93–8.09)	
SCLC	5.52 (3.2–7.84)	
Breast cancer	7.46 (1.89–13.03)	
Melanoma	9.53 (3.73–15.32)	
RCC	2.96 (1.73–4.18)	
other	4.17 (2.84–5.5)	
Diabetes mellitus		0.003
Yes	4.73 (2.81–6.65)	
No	6.7 (4.5–8.89)	
Arterial hypertension		0.443
Yes	5.95 (4.29–7.61)	
No	6.05 (3.51–8.58)	
Smoking history		0.307
Yes	6.08 (3.96–8.20)	
No	5.72 (3.67–7.77)	
PAOD		0.266
Yes	4.17 (0–9.3)	
No	5.95 (4.54–7.36)	
Hypercholesterolemia		0.157
Yes	4.11 (0–8.62)	
No	0.71 (4.65–7.45)	

**Table 3:** Patient characteristics in patients with/without diabetes mellitus.

Characteristic	*n*	With DM	Without DM	*p*-Value
Age (years)				0.027
<70	149	24 (61.5%)	125 (78.6%)	
≥70	49	15 (38.5%)	34 (21.4%)	
KPS				0.841
<70	51	10 (34.5%)	41 (32.5%)	
≥70	104	19 (65.5%)	85 (67.5%)	
Histology				0.606
NSCLC	84	20 (51,3%)	64 (40.3%)	
SCLC	20	4 (10%)	16 (10.1%)	
Melanoma	28	2 (5.1%)	26 (16.4%)	
Breast cancer	20	3 (7.7%)	17 (10.7%)	
Colorectal cancer	5	1 (2.6%)	4 (2.5%)	
RCC	19	5 (12.8%)	14 (8.8%)	
Other	22	4 (10.3%)	18 (11.3%)	
No. BM				0.792
≤3	77	16 (44.4%)	61 (46.9%)	
>3	69	20 (55.6%)	69 (53.1%)	
Stable systemic disease				0.154
Yes	27	2 (5.7%)	25 (19.4%)	
No	71	17 (48.6%)	54 (41.9%)	
Synchronous BM	66	16 (45.7%)	50 (38.8%)	
Radiation modality				0.441
SRT alone	40	6 (16.2%)	34 (21.9%)	
WBRT ± SRT	152	31 (83.8%)	121 (78.1%)	

## Data Availability

The datasets generated during and/or analyzed during the current study are available from the corresponding author on reasonable request.

## Supplementary Materials

The following supporting information can be downloaded at: https://www.mdpi.com/article/10.3390/cancers15194845/s1, Figure S1: Univariate and Multivariate analysis of survival (Cox-Regression) of patients with/without DM in patients with NSCLC, Table S1: Univariate and Multivariate analysis of survival (Cox-Regression) of prognostic factors in patients with NSCLC, Figure S2: Univariate and Multivariate analysis of survival (Cox-Regression) of patients with/without DM in patients with melanoma, Table S2: Univariate and Multivariate analysis of survival (Cox-Regression) of prognostic factors in patients with melanoma.
