# Supplementary material for: Diabetes Mellitus Is a Strong Independent Negative Prognostic Factor in Patients with Brain Metastases Treated with Radiotherapy
Source: Cancers (Basel). 2023 Oct 4;15(19):4845. doi: 10.3390/cancers15194845 (PMC10571851; doi:10.3390/cancers15194845)
Supplement: Supplementary file 1 [file cancers-15-04845-s001.zip › cancers-2514234-supplementary.pdf]

Supplements

Supplement Figure S1. Uni- and multivariate cox regression analysis of survival of patients with (red)/without (blue) DM in patients with NSCLC

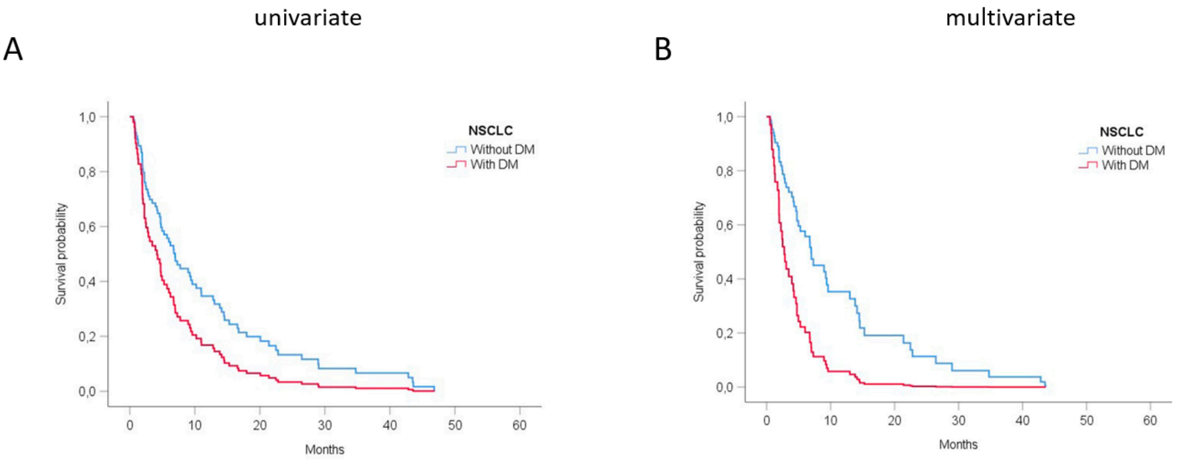

Supplement Table S1. Uni- and multivariate survival analysis (Cox-Regression) of prognostic factors in NSCLC patients

		Univariate analysis		Multivariate analysis	
		HR (95% CI)	p-value	HR (95% CI)	p-value
Diabetes mellitus	No	Ref		Ref	
	Yes	1.68 (0.97 – 2.93)	0.066	2.73 (1.32 – 5.63)	0.006
Age	<70	Ref		Ref	
	≥ 70	1.539 (0.85 – 2.72)	0.151	1.31 (0.63 – 2.73)	0.475
KPS	≥ 70	Ref		Ref	
	<70	2.47 (1.40 – 4.38)	0.002	2.85 (1.52 – 5.33)	0.001
No. of BM	≤ 3	Ref		Ref	
	>3	1.26 (0.77 – 2.04)	0.355	1.18 (0.67 – 2.10)	0.565

Supplement Figure S2. Uni- and multivariate cox regression analysis of survival of patients with (red)/without (blue) DM in patients with melanoma

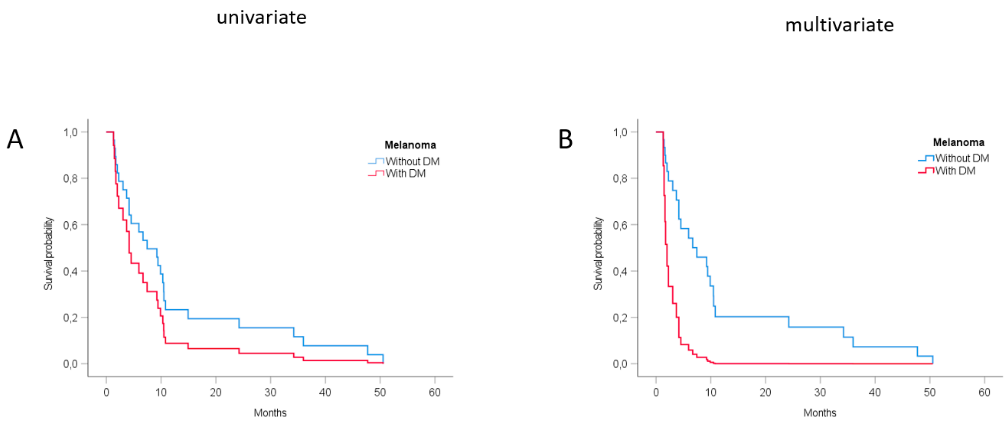

Supplement Table S2. Uni- and multivariate (Cox-Regression) of prognostic factors in melanoma patients

		Univariate analysis		Multivariate analysis	
		HR (95% CI)	p-value	HR (95% CI)	p-value
Diabetes mellitus	No	Ref		Ref	
	Yes	1.66 (0.38 – 7.27)	0.498	4.62 (0.49 – 44.04)	0.183
Age	<70	Ref		Ref	
	≥ 70	1.07(0.45 - 2.6)	0.875	0.98 (0.35 – 2.73)	0.966
KPS	≥ 70	Ref		Ref	
	<70	1.47 (0.6 – 3.61)	0.405	1.46 (0.55 – 3.93)	0.448
No. of BM	≤ 3	Ref		Ref	
	>3	1.27 (0.57 – 2.84)	0.555	1.18 (0.49– 2.81)	0.717
